# Evaluation of Anti-TBGL Antibody in the Diagnosis of Tuberculosis Patients in China

**DOI:** 10.1155/2015/834749

**Published:** 2015-08-03

**Authors:** Jingge Zhao, Zhaoqin Zhu, Xiaoyan Zhang, Yasuhiko Suzuki, Haorile Chagan-Yasutan, Haili Chen, Yanmin Wan, Jianqing Xu, Yugo Ashino, Toshio Hattori

**Affiliations:** ^1^Division of Emerging Infectious Diseases, Department of Internal Medicine, Graduate School of Medicine, Tohoku University, Sendai, Miyagi 980-8574, Japan; ^2^Shanghai Public Health Clinical Center, Fudan University, Shanghai 201508, China; ^3^Key laboratory of Medical Molecular Virology of the Ministries of Education, School of Basic Medical Science, Fudan University, Shanghai 201508, China; ^4^Division of Global Epidemiology, Hokkaido University Research Center for Zoonosis Control, Sapporo, Hokkaido 001-0020, Japan; ^5^Laboratory of Disaster-Related Infectious Disease, International Research Institute of Disaster Science, Tohoku University, Sendai, Miyagi 980-8574, Japan

## Abstract

Tuberculous glycolipid (TBGL) is a component of the *Mycobacterium tuberculosis* cell wall, and anti-TBGL antibodies are used for serodiagnosis of tuberculosis. Anti-TBGL IgG and IgA levels were measured in 45 pulmonary TB patients (PTB), 26 extra-pulmonary TB patients (ETB), 16 AIDS-TB patients, and 58 healthy controls (HC) including 39 health care workers (HW) and 19 newly enrolled students (ST). Anti-TBGL IgG measurements yielded 68.9% and 46.2% sensitivity in PTB and ETB, respectively, and 81.0% specificity. However, anti-TBGL IgA measurements were significantly less sensitive in detecting ETB than PTB (15.4% versus 46.7% sensitivity) but showed up to 89.7% specificity. Samples from AIDS-TB patients exhibited low reaction of anti-TBGL IgG and IgA with 6.3% and 12.5% sensitivity, respectively. Unlike anti-lipoarabinomannan (LAM) IgG that was found to elevate in sputum smearpositive subjects, anti-TBGL IgG and IgA elevated in those with cavitation and bronchiectasis, respectively. Anti-TBGL IgG in cavitary TB yielded 78.2% sensitivity compared to 57.1% in those otherwise. Meanwhile, higher anti-TBGL IgA titers were observed in HW than in ST, and increasing anti-TBGL IgG titers were observed in HW on follow-up. Therefore, higher anti-TBGL antibody titers are present in patients presenting cavities and bronchiectasis and subjects under TB exposure risk.

## 1. Introduction

In 2013, tuberculosis (TB) infected 9 million new individuals and caused 1.5 million deaths, making it one of the most critical infectious diseases worldwide. In terms of the number of reported cases, China ranks the second after India [1]. However, conventional microscopy is still widely used to diagnose TB, which renders variable sensitivities of 20–60% in detecting* tubercle bacilli* [2]. Moreover, approximately 20% of active TB cases and all latent TB infection (LTBI) cases cannot be microbiologically proven, even with fluorescence microscopy [3].

The specific IgG response to tuberculous glycolipid antigen (TBGL), a combination of trehalose-6,6′-dimycolate (TDM) and minor glycolipids, has been used to diagnose clinical TB infection in Japan, with approximately 80% sensitivity and specificity [4, 5]. The* WHO Stop TB Strategy* recommends TB screening and diagnostic algorithms should be implemented at a country level [6]. Previous studies performed in Japan have found that anti-TBGL IgG and anti-TBGL IgA titers correlate with cavitation and severities implicated by chest radiography [7]. Therefore, a combination of TBGL antibody detection and other TB clinical findings could further improve the accuracy of TB diagnosis [7, 8]. In a study in the Philippines, elevated anti-TBGL antibody titers were observed in healthcare workers (HW) with LTBI [9]. However, no study has evaluated anti-TBGL antibodies in TB patients or healthy individuals in China. Like TBGL, lipoarabinomannan (LAM), another glycolipid that constitutes mycobacterial cell walls, has been immensely investigated for its important roles in the immune-pathogenesis of TB [8–10], albeit limitations of anti-LAM IgG in TB diagnosis that may mislead diagnosis in about a quarter of cases [10]. Therefore, it is important to evaluate anti-TBGL antibodies in the context of varied TB pathogenesis attributable to pulmonary TB (PTB) patients, extrapulmonary TB (ETB) patients, and AIDS-TB patients.

## 2. Materials and Methods

### 2.1. Study Subjects

Blood samples were drawn from adult subjects (age > 18) recruited from Shanghai Public Health Clinical Center (SHAPHC), affiliated with Fudan University, Shanghai, China, between 2008 and 2011, after informed consent was obtained according to the protocol approved by the ethics committees from SHAPHC and the Tohoku University School of Medicine, Japan (20121322). PTB, ETB, and AIDS-TB samples were obtained from hospitalized patients who had undergone less than 2 weeks of anti-TB treatment. Blood was drawn after diagnosis of TB (Table 1). ETB samples included samples from patients with 9 different subtypes of ETB (Table 2). HIV-1 infection in the AIDS-TB patients was confirmed by detecting HIV-1 antibodies. The healthy control subjects (HC) recruited included 19 students (ST) and 39 health care workers (HW). The samples used for follow-up analysis were collected from 16 HW who underwent an annual checkup from 2009 to 2011. All HC subjects were diagnosed as free from active TB based on chest radiography and free from HIV infection at the time of blood collection.

### 2.2. Anti-TBGL Antibodies

**Figure 1:**
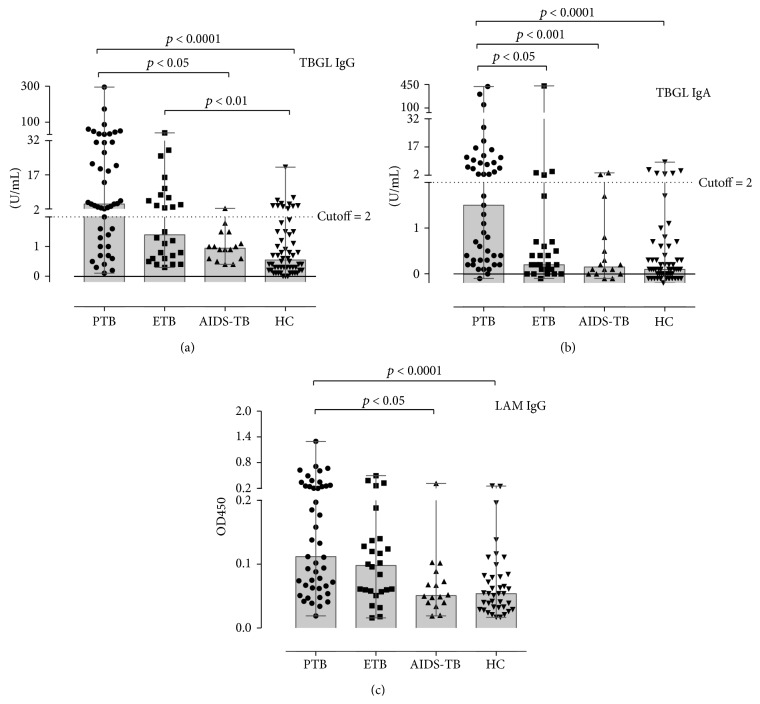
Detection of anti-TBGL IgG, anti-TBGL IgA, and anti-LAM IgG in TB patients and HC. PTB = pulmonary TB; ETB = extrapulmonary TB; HC = healthy control. Anti-TBGL antibody titers were evaluated in U/mL, and the anti-LAM IgG reactivity was assessed as OD values.

Plasma levels of anti-TBGL IgG and IgA were measured using a Determiner TBGL Antibody ELISA kit (Kyowa Medex Co., Ltd., Tokyo, Japan). This assay uses TBGL antigen which is a combination of trehalose 6,6-dimycolate (TDM) and hydrophobic glycolipids extracted from MTB H37Rv and has been described previously [7]. The cutoff index for anti-TBGL IgG and IgA was set to 2.0 U/mL in accordance with previous studies (Figure 1(a)) [4, 5].

### 2.3. Anti-LAM IgG ELISA

The anti-LAM IgG ELISA method has been previously described [11]. ELISA Nunc MaxiSorp plates (Thermo Fisher Scientific, Inc., Waltham, MA) [12] were coated with 100 *μ*L per well of 0.5 *μ*g/mL purified lipoarabinomannan (LAM) (NACALAI TESQUE, INC.). A polyclonal antibody from Dr. Makoto Matsumoto (Otsuka Pharmaceutical Co., Ltd., Tokushima, Japan), which was made from a LAM immunized rabbit, was used as a positive control. Serum samples were diluted 100-fold in fetal bovine serum and incubated in coated well for 1 hour. After being washed, HRP-conjugated goat anti-human IgG heavy chain polyclonal antibody (LifeSpan BioSciences, Inc., Seattle, WA) was diluted 1 : 10,000 in 1% (w/v) BSA in PBS and added as the secondary antibody to detect anti-LAM IgG. Reactions were visualized using a TMB HRP substrate kit (KPL, Inc., Gaithersburg, MD). Optical density (OD) values were measured at 450 nm. The cutoff for anti-LAM IgG was set based on Receiver Operating Characteristic (ROC) curve (Figure 3(c)).

### 2.4. Statistical Analysis

All data were analyzed using GraphPad Prism 6.0 (GraphPad Software, San Diego, CA). A nonparametric *t*-test was used to determine the significance of differences between 2 groups with non-Gaussian distributions.* Kruskal-Wallis* tests were used to evaluate differences when more than 2 groups were involved.* Dunn's post hoc* tests were used to evaluate the differences between 2 groups following the* Kruskal-Wallis* test. A Chi-square test was used to test the variances among groups. The results were considered significant at *p* < 0.05. Paired nonparametric *t*-tests were used to compare the differences in the same samples in the three years of follow-up. Estimated optimal cutoff for other antibodies was achieved by Youden's index by MedCalc (MedCalc Software bvba, Belgium).

## 3. Results

### 3.1. Anti-TBGL Antibodies and Anti-LAM Antibodies

For anti-TBGL IgG, more samples from PTB patients had elevated titers compared to those with AIDS-TB (*p* < 0.05) or compared to HC (*p* < 0.0001). Amongst ETB samples, more samples had elevated titers of anti-TBGL IgG compared to HC (*p* < 0.01) (Figure 1(a)). Of note, there was no difference in anti-TBGL IgG titers between PTB and ETP samples. For anti-TBGL IgA, significantly higher titers were observed in the PTB samples compared to those of other groups (*p* < 0.0001 for HC, *p* < 0.001 for AIDS-TB, and *p* < 0.05 for ETB). However, there was no difference between ETP and HC samples or between ETP and AIDS-TB samples (Figure 1(b)). For anti-LAM IgG, significantly larger number of samples with higher responses was found amongst those from PTB patients compared to HC (*p* < 0.001) or AIDS-TB (*p* < 0.05) patients. Nevertheless, measurements of ETB samples were not significantly different from those of HC or AIDS-TB samples for anti-LAM IgG. Unlike that for anti-TBGL IgA, no difference was observed between the PTB and ETB samples with respect to anti-LAM IgG (Figure 1(c)).

### 3.2. Serodiagnosis amongst TB Patients

**Figure 2:**
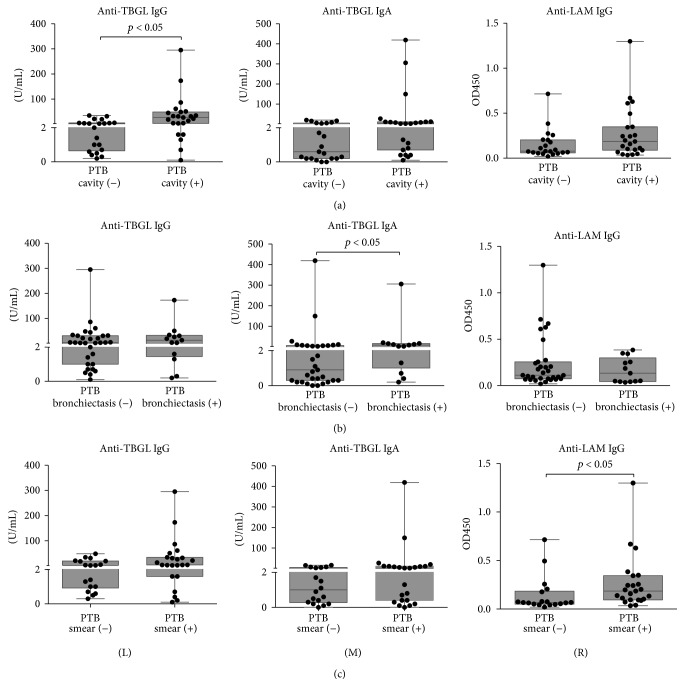
Elevated titers in association with different clinical findings. (a), (b), and (c) display findings of PTB-cavity versus PTB-noncavity, PTB-bronchiectasis versus PTB-nonbronchiectasis, and PTB-smear-negative versus PTB-smear-positive, respectively. A *p* value less than 0.05 indicates a significant difference between 2 groups by nonparametric *t*-test. (L), (M), and (R) referred to anti-TBGL IgG, anti-TBGL IgA, and anti-LAM IgG, respectively.

**Figure 3:**
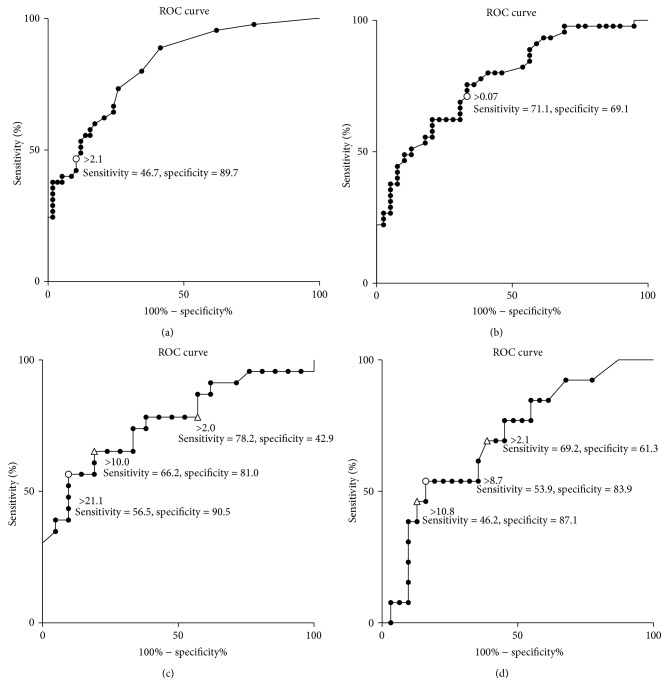
ROC analysis for different antibodies. (a) and (b) ROC analysis between PTB patients and HC for anti-TBGL IgA and anti-LAM IgG, respectively; (c) ROC analysis between cavities positive and negative subjects for anti-TBGL IgG; (d) ROC analysis between bronchiectasis positive and negative subjects for anti-TBGL IgA.* Circle*: estimated optimal cutoff according to Youden's index;* triangle*: arbitrary cutoff.

PTB patients were grouped in correspondence with the clinical findings listed in Table 1. Anti-TBGL IgG titers were significantly higher in patients with cavitation compared to those without such pathology, and significantly elevated anti-TBGL IgA titers were observed in subjects with bronchiectasis compared to those without it (*t*-test, *p* < 0.05, Figures 2(a) and 2(b)). However, there were no differences between subgroups for anti-LAM IgG with respect to chest radiographic findings (*p* > 0.05, Figure 2(c)), in spite of the fact that there were subjects with positive sputum smears that were higher anti-LAM IgG responses, which was not observed for anti-TBGL IgG or IgA (Figure 2(c)). Similar to PTB patients, ETB patients showed relatively high anti-TBGL IgG titers in contrast to anti-TBGL IgA titers which were lower in ETB samples. Remarkably, all 3 intestinal TB subjects were anti-TBGL IgG positive (Median 15.9 Range [3.9–27.9]), while only 2 out of 7 TB pleurisy subjects showed positive in anti-TBGL IgG (Median 1.2 Range [0.4–7.0]). Samples from AIDS-TB patients had considerably lower titers for anti-TBGL antibodies compared to other TB groups (Figures 1(a) and 1(b)).

### 3.3. Anti-TBGL Antibody and Anti-LAM IgG in Healthy Controls (HC)

**Figure 4:**
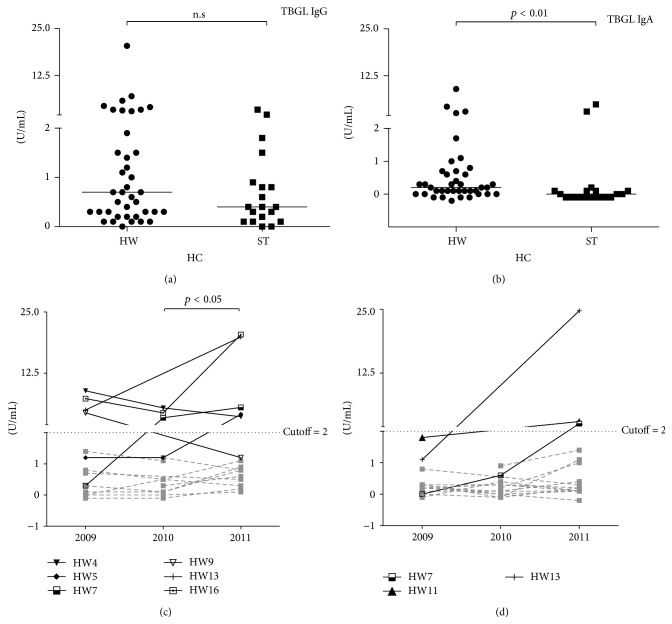
Detection of anti-TBGL antibodies and anti-LAM IgG among HC including HW and ST and follow-up study of HW. HW refers to health care workers; ST refers to new enrolled students (a and b); 2009, 2010, and 2011 refer to different years of sample collection (c and d).

We examined HC and HW anti-TBGL antibody titers. There are two subgroups of HC in this study, HW who worked in environments with higher risk to TB exposure and ST who had merely enrolled less than 1 year in the lab. The age of HW (30.51 ± 0.99, *n* = 39) was significantly higher than that of ST (24.16 ± 1.38, *n* = 19) (Table 1). Age did not correlate with either anti-TBGL antibody or anti-LAM IgG titers within HC or ST (Spearman, *p* > 0.05). The difference between ST and HW for anti-TBGL IgG and anti-LAM IgG titers was not statistically significant (nonparametric *t*-test, *p* > 0.05 for both), while such difference for anti-TBGL IgA titers was significant (*p* < 0.01, Figure 4(b)). We also performed a follow-up study of anti-TBGL antibodies in 16 HW. In the three-year follow-up, we observed a trend of HW subjects with increasing positive for anti-TBGL IgG but not for anti-TBGL IgA (*p* < 0.05, Figures 4(c) and 4(d)).

## 4. Discussion

The findings that the highest levels of anti-TBGL IgG were associated with cavitation in Chinese PTB patients were consistent with a previous study on Japanese subjects [7], a trend that was not observed for anti-LAM IgG. In contrast, higher anti-LAM IgG responses were observed in patients who are sputum smear positive, in agreement with a previous study [10, 13]. LAM-Anti-LAM complex may be formed in the serum of the sputum negative samples, reducing the sensitivity of anti-LAM antibody detection [14]. On the other hand, LAM was found more frequently in the urine of smear-positive patients, suggesting a constant stimulus for anti-LAM antibodies existing in such patients [15]. LAM may not associate with cavitation, while TBGL contained TDM, a cord factor, which has been reported to induce cavitation and granulomatous responses [16, 17]. CD1d targets TDM, which may play a critical role in hypersensitive granulomatous response to mycobacterial cord factor [18]. Therefore, in spite of the elevated titers of anti-TBGL IgG and anti-LAM IgG in PTB patients (Figures 1(a) and 1(c)), results of the clinical factor analysis for PTB suggested that anti-TBGL antibody responses are associated with a pathogenesis different from that associated with anti-LAM antibody. Although anti-TBGL IgA showed a higher titer in bronchiectasis PTB patients, 9 out of 13 patients presented with cavitation. In China, TB antibody measurements serving as a suggestive reference must be in combination with a third TB specific clinical reference for TB diagnosis. Amongst clinical findings, smear positive appears less challenging compared to cavitation and bronchiectasis to diagnose TB [19] that can be found not only in TB but also in other lung infections, such as* Aspergillus fumigatus* infection, or in lung cancer; therefore measurement of anti-TBGL antibody may benefit confirmation of PTB infection with cavitation or bronchiectasis radiography and avoid being compromised by sputum smear results [5, 8].

Elevated anti-TBGL IgG titers but low anti-TBGL IgA and anti-LAM IgG titers were observed in ETB patients (Figures 1(a) and 1(b)). Although higher anti-TBGL IgG titers were detected in all the ETB subtypes combined, anti-TBGL IgG titers varied with different sites of ETB. Of note, tuberculous pleurisy showed relatively lower anti-TBGL IgG titers as compared to intestinal TB (*p* < 0.05, data not shown), suggesting an association between anti-TBGL antibody and specific sites of ETB where gastrointestinal mucosal may participate in responding to TBGL [20], as anti-TBGL antibody titer was found to decrease significantly in gastrectomized TB patients in our previous study [21]. Low titers of TB related antibody have been found in TB pleurisy in previous studies, but the reason is still unclear [22]. Low anti-TBGL antibodies and anti-LAM IgG titers were observed in AIDS-TB patients (Figure 1) due to failure in the development of potent humoral responses [23] and a malfunctioning gastrointestinal immune system [24].

The possibility of health care workers developing latent TB infection (LTBI) was 2.02–2.76 times higher than that of those in low-risk workplaces, and the LTBI incidence rate in HW was reported to be up to 43.4% in China [25]. In our previous study in the Philippines, we found elevated titers of both anti-TBGL IgG and IgA in HC subjects with diagnosed LTBI [9]. Of note, the follow-up study on 16 HW showed significant increase in anti-TBGL IgG titers over 3 years in the current study, though the significance of the data is not clear because LTBI was not confirmed.

The strength of this study, performed in a TB-prevalent country, is that anti-TBGL antibody detection could be sensitive and specific for PTB and ETB patients and HC in the context of clinical manifestation. In PTB, high anti-TBGL IgG and IgA titers were found to be associated with cavitation and bronchiectasis, respectively. In ETB, elevated anti-TBGL IgG titer in the absence of anti-TBGL IgA titer may serve as a pair of biomarkers to diagnose ETB from PTB. TBGL antibody titers may be high in those who have been at risk of TB exposure, thereby raising cautions for low sensitivity of anti-TBGL antibodies test for healthy individuals. The limitation of this study includes the lack of the prognostic observation for PTB subjects, insufficient samples from each subgroup of ETB, and the inability to confirm LTBI infection in HW.

## Figures and Tables

**Table 1:** Clinical and demographic information of study patients.

Parameter	Value for group			
	PTB	ETB	AIDS-TB	HC
Number of patients	45	26	16	58
Age (years [mean ± SD])	60.47 ± 18.19	46.83 ± 20.30	45.53 ± 8.02	28.43 ± 5.92
Sex (number of males/number of females)	35/10	11/15	12/3	41/17
Classification (number)				
New case	28	21	12	0
Relapsed case	11	4	3	0
Unknown	6	1	1	0
Comorbidities^a^				
Chronic diseases	27/17 (61.4)	9/17 (34.6)	5/9 (33.3)	n.a
Symptoms^ a^				
Cough & chest discomfort	38/6 (86.4)	12/12 (50.0)	8/7 (53.3)	0/58 (0.0)
Fever	14/30 (31.8)	15/9 (62.5)	11/4 (73.3)	0/58 (0.0)
Hemoptysis	12/32 (27.3)	1/23 (4.2)	0/16 (0.0)	0/58 (0.0)
Expectoration	23/21 (52.3)	5/20 (20.0)	0/16 (0.0)	0/58 (0.0)
Chest X-ray^ a^				
Cavitation	24/20 (54.5)	0/26 (0.0)	0/16 (0.0)	0/58 (0.0)
Pleural effusion	15/29 (34.1)	10/15 (40.0)	5/9 (35.7)	0/58 (0.0)
Lymphadenopathy (BHL)	3/41 (6.8)	3/22 (12.0)	3/11 (21.4)	0/58 (0.0)
Bronchiectasis	13/31 (29.5)	2/23 (8.0)	0/14 (0.0)	0/58 (0.0)
TB screening test^ a^				
Smear	23/18 (56.1)	7/14 (33.3)	9/4 (69.2)	0/58 (0.0)
TST	11/6 (64.7)	7/3 (70.0)	1/0 (100.0)	0/58 (0.0)
TB antibody^ a^				
Anti-TBGL IgG^b,c,e^	31/14 (68.9)	12/14 (46.2)	1/15 (6.3)	11/47 (19.0)
Anti-TBGL IgA^b,c,d^	21/23 (46.7)	4/22 (15.4)	2/14 (12.5)	6/52 (10.3)
Others mean ± std				
Blood IgA (g/L)	3.41 ± 1.76	3.13 ± 0.75	n.a	n.a
Blood IgG (g/L)	14.82 ± 4.64	16.50 ± 2.84	n.a	n.a
Blood IgM (g/L)	1.11 ± 0.50	1.49 ± 0.62	n.a	n.a
CRP (mg/L)	26.07 ± 27.64	28.95 ± 26.87	n.a	n.a
CD4 count	n.a	n.a	159.75 ± 177.16	n.a
CD8 count	n.a	n.a	636.38 ± 438.26	n.a

PTB = pulmonary TB; ETB = extrapulmonary TB; HC = healthy control. ^a^The number of positive subjects/the number of negative subjects (percentage of positive subjects). ^b^Significant difference between PTB and HC samples (*p* < 0.05). ^c^Significant difference between PTB and AIDS-TB samples (*p* < 0.05). ^d^Significant difference between PTB and ETB samples (*p* < 0.05). ^e^Significant difference between ETB and HC samples (*p* < 0.05).

**Table 2:** Anti-TBGL IgG, anti-TBGL IgA, and anti-LAM IgG in ETB patients.

Subtypes of ETB (*n* = 26)	Number	TB related Biomarkers					
		Anti-TBGL IgG		Anti-TBGL IgA		Anti-LAM IgG	
Tuberculous pleurisy	7	2 (28.6%)^a^	1.2 [0.4–7.0]^b^	1 (14.3%)	0.4 [0–4.0]	2 (28.6%)	0.06 [0.02–0.50]
Tuberculous meningitis	4	1 (25%)	0.65 [0.6–8.2]	0 (0%)	0.05 [−0.1–0.7]	1 (25%)	0.05 [0.03–0.08]
Miliary TB	3	2 (66.7%)	2.9 [1.3–5.6]	1 (33.3%)	0.6 [0–2.1]	3 (100%)	0.10 [0.01–0.14]
Intestinal TB	3	3 (100%)	15.9 [3.9–27.9]	1 (33.3%)	1.7 [0.2–428.8]	3 (100%)	0.14 [0.12–0.33]
Lymph node TB	3	1 (33.3%)	0.5 [0.4–11]	0 (0%)	0.2 [0.2–0.2]	2 (66.7%)	0.12 [0.06–0.39]
Renal TB	2	1 (50%)	20.4 [0.3–40.5]	0 (0%)	0.45 [0.2–0.7]	1 (50%)	0.12 [0.06–0.19]
Bone TB	2	1 (50%)	12.85 [0.4–25.3]	0 (0%)	0.2 [0–0.4]	2 (100%)	0.11 [0.10–0.12]
Endometrial TB	1	1 (100%)	3.5 [3.5]	1 (100%)	3.3 [3.3]	0 (0%)	0.06 [0.06]
Extrarenal TB	1	0 (0%)	0.8 [0.8]	0 (0%)	0.1 [0.1]	1 (100%)	0.13 [0.13]

ETB = extrapulmonary TB. ^a^positive number (percentage) in compariso with the cut off of anti-TBGL IgG (cut off = 2), anti-TBGL IgA (cut off = 2), and anti-LAM IgG (cut off = 0.07), respectively; ^b^median [range].
